# The Invasive Plant *Impatiens glandulifera* Manipulates Microbial Associates of Competing Native Species

**DOI:** 10.3390/plants12071552

**Published:** 2023-04-04

**Authors:** Nadia Ab Razak, Alan C. Gange, Brian C. Sutton, Asyraf Mansor

**Affiliations:** 1 Centre for Chemical Biology, Sains@USM, Universiti Sains Malaysia, Pulau Pinang 11900, Malaysia; 2 Department of Biological Sciences, Royal Holloway University of London, Egham TW20 0EX, Surrey, UK; a.gange@rhul.ac.uk (A.C.G.); briansutton512@btinternet.com (B.C.S.); 3 School of Biological Sciences, Universiti Sains Malaysia, Pulau Pinang 11800, Malaysia

**Keywords:** arbuscular mycorrhizal fungi, invasive species, native species, plant competition, insect, endophyte

## Abstract

*Impatiens glandulifera* or Himalayan balsam is one of the most invasive weeds across Europe and can seriously reduce native plant diversity. It often forms continuous monocultures along river banks, but the mechanisms of this arrested succession are largely unknown. Here, we investigated the effect of arbuscular mycorrhizal (AM) fungi on balsam competitive ability with two native plant species, *Plantago lanceolata* and *Holcus lanatus*. We also studied how competition with *Impatiens* affects colonisation by foliar endophytes and mycorrhizas of two other co-occurring native species, *Urtica dioica* and *Cirsium arvense.* Mycorrhizal colonisation reduced balsam growth when the plants were grown singly, but appeared to have little effect when balsam experienced intra- or interspecific competition. Competition with balsam together with the addition of mycorrhizas had no effect on *P. lanceolata* biomass, suggesting that the fungi were beneficial to the latter, enabling it to compete effectively with balsam. However, this was not so with *H. lanatus.* Meanwhile, competition with *Impatiens* reduced endophyte numbers and mycorrhizal colonisation in *U. dioica* and *C. arvense,* leading to enhanced susceptibility of these plants to insect attack. Himalayan balsam is known to degrade soil fungal populations and can also reduce foliar beneficial fungi in neighbouring plants. This allows the plant to compete effectively with itself and other native species, thereby leading to the continuous monocultures.

## 1. Introduction

Numerous factors contribute to the invasion success of *Impatiens glandulifera.* It can grow rapidly up to 4 m tall, making it the tallest annual herb in Europe. Because it is much taller than native herbaceous species, it is a strong competitor [1]. As one would expect from a large plant, the seed production is extremely high (up to 2500 per plant and 30,000 m^−2^) and efficiently dispersed (up to 7 m), aided by a ballistic mechanism [2]. These characteristics could make it a more significant threat to the environment in the future [3,4] with range expansion likely under future climate warning scenarios [5]. Himalayan balsam markedly outcompeted native species *Salix alba* and *Urtica dioica* through its superior growth rate [6]. Additionally, it outperformed other native species (*Agrostis stolonifera, Populus nigra* and *Rubus caesius*) as well as four other invasive species (*Acer negundo, Buddleja davidii, Fallopia japonica*, and *Paspalum distichum*) in interspecific competition studies [6]. Interestingly, despite being the most sensitive to disturbance [7], *I. glandulifera* was still able to outcompete the perennial herbaceous native species, *Urtica dioica* [6,8]. It was suggested that the strong allelopathic effect of *I. glandulifera* on native species served to promote its superior competitive ability [8]. Furthermore, by demonstrating positive plant–soil feedback over the course of several growing seasons, *I. glandulifera* can become an aggressive and better competitor with other species in self-replicating stands [9], mainly through the degradation of soil microbial communities, particularly arbuscular mycorrhizal fungi (AMF). These fungi appear to antagonise growth of *I. glandulifera,* unlike in many invasive plants, where AMF enhance invasion success, reducing native diversity through competitive effects [10]. 

Plant competition between invasive plants and native vegetation can be mediated by arbuscular mycorrhizal (AM) fungi [11], determining the structure of plant communities [12]. However, the identity of the mycorrhizal species, the competing plants, and whether they are AM fungi-dependent or not all have an impact on plant competition, in addition to environmental conditions [13,14]. For example, AM fungi strongly enabled *Centaurea maculosa* (Spotted knapweed), a mycorrhizal-dependent invasive plant species, to compete with *Festuca idahoensis* in western North America [15]. Indeed, these fungi have been implicated in the success of many strongly mycorrhizal invasive species in the Asteraceae [16]. However, AM fungal colonisation can reduce *I. glandulifera* growth [17,18], and it is unclear how mycorrhizas affect competition between *I glandulifera* and native plants. Unravelling these mechanisms is critical to understanding why *I. glandulifera* forms continuous monocultures and affects native plant diversity. 

Such interactions are important and can determine the extent of invasion success. For example, when the two species were grown separately, mycorrhizal colonisation increased shoot biomass in the deciduous shrub *Acacia caven* while decreasing it in the invasive annual forb *Bidens pilosa.* However, these effects vanished when the plants were grown at higher densities [19]. This suggests that there is an interaction between mycorrhizal colonisation in the roots and plant density and the existence of shared mycelial networks [20], which ultimately affect the outcome of plant competition and invasion success. How mycorrhizal colonisation affects the outcome of plant interspecific competition depends to a large extent on the mycorrhizal affinity of the dominant and sub-dominant species. If the dominant is strongly mycorrhizal, AM fungi enhance this dominance, leading to reduced species diversity, while if the sub-dominants are strongly mycorrhizal, AM fungi enhance their growth, leading to increased species diversity [11]. However, the outcome is far less clear if the dominant is antagonised by AM fungi, as in the case with *I. glandulifera.* With intraspecific competition, AM fungi increase the intensity of competition in strongly mycorrhizal species [21], but there are very few studies that compare AM fungal effects in species that are strongly mycorrhizal with those antagonised by AM fungi. The only study to date concerns two tropical tree species, in which AM fungi enhanced competitive effects in the mycotrophic species, but relaxed these in the species where fungi were parasitic [22]. It is therefore important to understand how AM fungi affect intraspecific competition in *I. glandulifera*, given that the species frequently occurs in populations where intraspecific competition is intense.

Unlike mycorrhizal fungi, very little is known about how foliar endophyte fungi influence the success of invasive forbs [23], though clavicipitaceous endophytes are often implicated in the invasion success of grasses [24]. Indeed, one recent example suggests that endophytes have facilitated the invasion success of the annual grass *Poa annua* in Antarctica [25]. This knowledge gap is despite the fact that Evans [26] proposed the endophyte–enemy release hypothesis, in which it was postulated that invasive plants may be introduced with their co-evolved endophytes, giving them protection against any local natural enemies, leading to enhanced competitive success [27]. Furthermore, endophytes can enhance the allelopathic effects of invasive species [28], which could be an important factor influencing the outcome of competition between *I. glandulifera* and native species. 

Although unspecialised endophytes (described and defined in [29]) are ubiquitous, there are plant-species-specific differences in community composition even between neighbouring plants [30]. Recently, it has been found that endophytes infecting *I. glandulifera* can afford some protection against the pathogenic fungus *Puccinia komarovii* var. *glanduliferae* introduced as a biological control agent [31], which may partly explain why the success of this introduction in the UK has been inconsistent [32]. These endophytes can also afford protection against antagonists in a wide range of plants [33]; however, whether the presence of any one plant species can affect the endophyte composition of a neighbour is unknown. Given that *I. glandulifera* can reduce the size of neighbours through direct competitive effects or allelopathic chemicals [34], it is possible that it may reduce endophyte infection in co-occurring native species, rendering them more susceptible to antagonists. If so, this would represent a novel mechanism underpinning the competitive nature and invasion success of an introduced plant. 

Here we report on three studies, investigating how the microbial associates of *I. glandulifera* influence competition of this plant with native species. We first examined how the presence of AM fungi influence growth of *I. glandulifera* when it experienced intraspecific competition, or interspecific competition with two common co-occurring species, *Plantago lanceolata* and *Holcus lanatus.* We also studied whether AM fungi and balsam presence could influence endophyte abundance in these latter species. We hypothesised that AM fungal colonisation would weaken balsam growth while enhancing that of the competing native species, resulting in smaller plants of *I. glandulifera* when mycorrhizas were present. Our second study involved recording mycorrhizal and endophyte abundance in two other common co-occurring plants, *Urtica dioica* and *Cirsium arvense,* when these plants were growing adjacent to or distant from *I. glandulifera.* Here we hypothesised that proximity to balsam would reduce AM colonisation in the neighbours (through soil microbial degradation) and would reduce endophyte abundance through competitive effects on neighbouring plant size. Thirdly, we conducted a controlled field experiment in which *U. dioica* and *C. arvense* were grown with or without AMF in pots, with or without *I. glandulifera.* To measure plant resistance, leaves of our target species were fed to larvae of a generalist insect at the end of this study. We hypothesised that the presence of AM fungi and balsam would reduce endophyte infection, leading to increased herbivore growth [29]. Finally, we hypothesised that mycorrhizal presence would reduce insect growth [35], but this would also depend on the presence of balsam. 

## 2. Results

### 2.1. Experiment One—Effect of Direct Competition with I. glandulifera on Two Native Species

**Figure 1:**
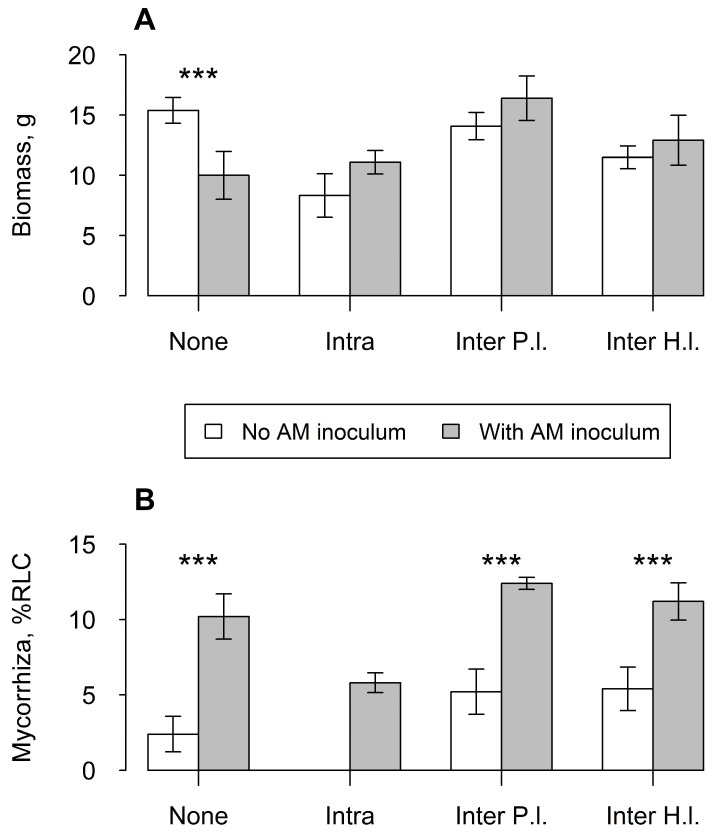
Mean of (**A**) dry shoot biomass and (**B**) AM fungal colonisation of *Impatiens glandulifera* across treatments. ‘None’ indicates a single balsam with no competition as a control. ‘Intra’ was intraspecific competition while ‘Inter P.l.’ and ‘Inter H.l.’ were interspecific competition with *Plantago lanceolata* and *Holcus lanatus*, respectively. %RLC: Percentage of root length colonised. Vertical bars represent ± one standard error. Asterisks above bars indicate significance of difference between means, ***: *p* < 0.001.

Addition of mycorrhizal inoculum increased the growth of *I. glandulifera* when in competition (*F*_1,36_ = 8.706, *p* < 0.001). The fact that the addition of mycorrhizal fungi tended to reduce the biomass of *I. glandulifera* when grown singly but increased it when growing in competition was perhaps most intriguing (Figure 1A). Although some colonisation was observed in uninoculated plants, the addition of mycorrhizal inoculum significantly increased colonisation levels of *I. glandulifera* (*F*_1,36_ = 38.155, *p* < 0.001) (Figure 1B). Single *I. glandulifera* plants had colonisation levels comparable to plants in the interspecific competition treatments, but twice as high as plants experiencing intraspecific competition (*F*_1,36_ = 6.269, *p* < 0.001). No colonisation was found in plants receiving sterilised inoculum and experiencing intraspecific competition (Figure 1B). 

**Figure 2:**
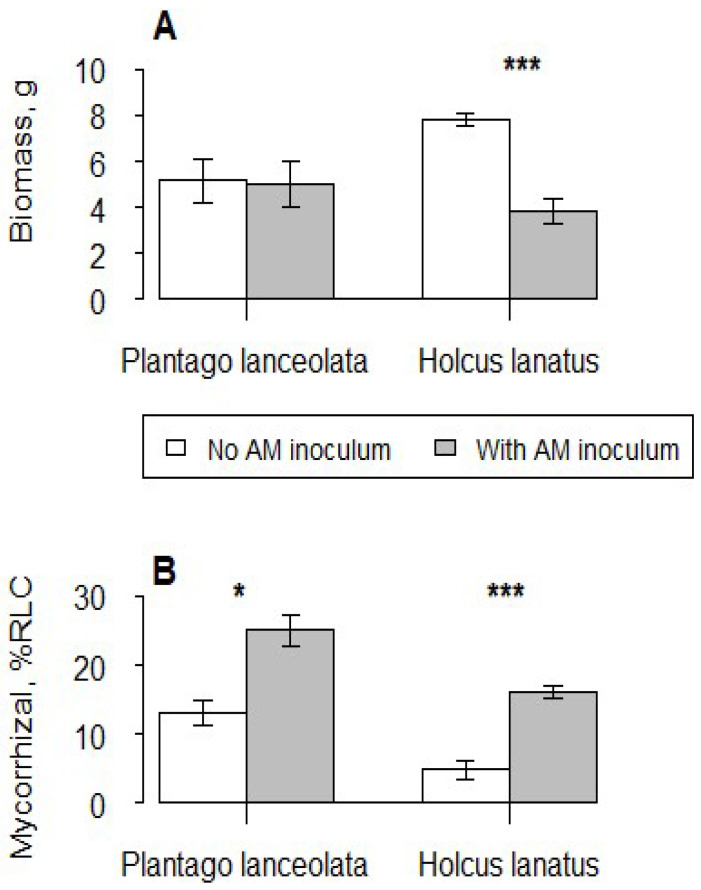
Mean of (**A**) dry shoot biomass and (**B**) AM fungal colonisation of the native plant species when grown in interspecific competition with *I. glandulifera*. Vertical bars represent ± one standard error. Asterisks above bars indicate significance of difference between means, *: *p* < 0.05; ***: *p* < 0.001.

When grown in competition with *I. glandulifera, P. lanceolata* biomass was unaffected by mycorrhizal addition, but *H. lanatus* biomass was reduced by the addition of AM fungi (*F*_1,8_ = 22.71, *p* < 0.001) (Figure 2A). Some colonisation was also observed in uninoculated plants of the native species, but both *P. lanceolata* (*F*_1,8_ = 10.45, *p* < 0.05) and *H. lanatus* (*F*_1,8_ = 16.52, *p* < 0.001) colonisation levels were increased by the addition of mycorrhizal inoculum (Figure 2B). 

**Figure 3:**
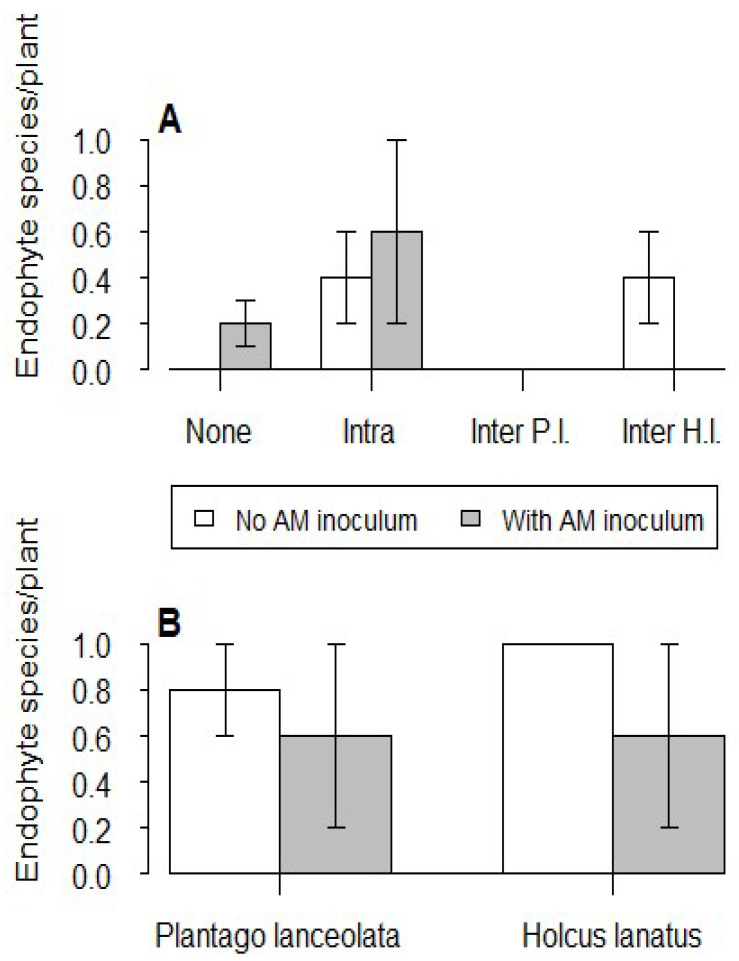
(**A**): Mean number of endophyte fungal species per plant in *I. glandulifera* when grown singly (None), experiencing intraspecific competition (Intra), or experiencing interspecific competition with *P. lanceolata* (Inter P.l.) or *H. lanatus* (Inter H.l.). (**B**): Mean number of endophyte species per plant in *P. lanceolata* and *H. lanatus* when grown in competition with *I. glandulifera.* Vertical bars represent ± one standard error.

Endophyte fungi were of sporadic occurrence in this experiment, and the addition of mycorrhizal fungi had no effect on the number of species per plant in any of the three species (Figure 3). Plants of *I. glandulifera* experiencing intraspecific competition tended to yield the most endophytes, while plants competing with *P. lanceolata* failed to yield any fungi (Figure 3A). Endophyte species showed high inter-plant variability in both *P. lanceolata* and *H. lanatus,* with no fungi being recovered from several plants (Figure 3B). As a result, there was no effect of AM fungal addition on endophyte numbers in these two species. 

*Cladosporium oxysporum* was the most prevalent of the seven endophyte species found in *I. glandulifera* (Table S1), while *Exophiala* spp. was the most prevalent of the five endophyte species found in native plant species (Table S2). Himalayan balsam that grew by itself produced only one species (*C. oxysporum)*, whereas *I. glandulifera* that competed with *P. lanceolata* produced none. When competing with *H. lanatus,* a total of three species were found in *I. glandulifera*, but only when AM fungi were absent. After adding mycorrhizal inoculum, no endophytes were found (Figure 3A). It is worth noting that two endophyte species (*Acremonium incoloratum* and *Alternaria alternata*) were found in intraspecific competition when mycorrhizal inoculum was used, but not in other treatments. In addition, *C. oxysporum* was found in *P. lanceolata* regardless of the presence of mycorrhizal fungi. Similar to *H. lanatus*, *Exophiala* spp. was isolated whether or not AM fungi were present. There was no difference in the isolation frequency mean of each endophyte fungal species and no difference in the species richness of endophyte fungal communities across the treatments.

### 2.2. Experiment Two—I. glandulifera Effects on Microbial Associates of the Native Species

**Figure 4:**
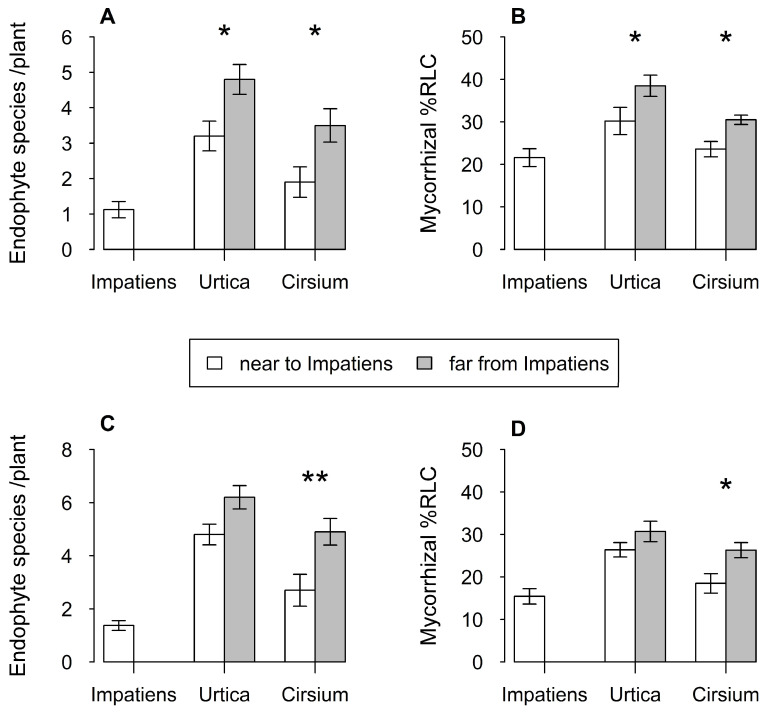
Mean number of endophyte fungal species per plant and percent root length colonised (RLC) by AM fungi of *Urtica dioica* and *Cirsium arvense* in two localities; Royal Holloway (**A**,**B**) and Zeals (**C**,**D**). White bars indicate plants growing interspersed with *I. glandulifera* stands, grey bars indicate plants over 100 m from these stands. The mean values for *I. glandulifera* are also given for each locality. Vertical lines represent ± one standard error. Asterisks above bars indicate significance of difference between means, *: *p* < 0.05; **: *p* < 0.01.

Plants of both *U. dioica* and *C. arvense* contained fewer endophyte fungal species when growing in close proximity to *I. glandulifera* at Royal Holloway (z = 6.5, df = 1, *p* < 0.05 and z = 5.5, df = 1, *p* < 0.05, respectively) (Figure 4A). A similar result was found at Zeals (z = 5.1, df = 1, *p* < 0.05 and z = 6.1, df = 1, *p* < 0.05, respectively) (Figure 4C). Plants of *I. glandulifera* contained fewer fungal species than either of the two focal plants in both localities. Colonisation levels of *I. glandulifera* by AM fungi were more similar to those of the two focal species (Figure 4B,D), and while there was a trend for colonisation to be lower in both species when growing close to *I. glandulifera*, this was only significant for *C. arvense* (Royal Holloway: *F*_1,18_ = 9.6, *p* < 0.01; Zeals: *F*_1,18_ = 6.3, *p* < 0.05) (Figure 4B,D).

Lists of all endophyte species found in the three plant species in each locality are given in Tables S3 and S4. Perhaps the most interesting species was *Colletotrichum acutatum.* This species was found in *I. glandulifera* in both localities and also within *U. dioica* and *C. arvense*, but only when these plants were growing at a distance from *I. glandulifera.*

### 2.3. Experiment Three—Effect of Competition with I. glandulifera on Plant Microbial Associates

**Figure 5:**
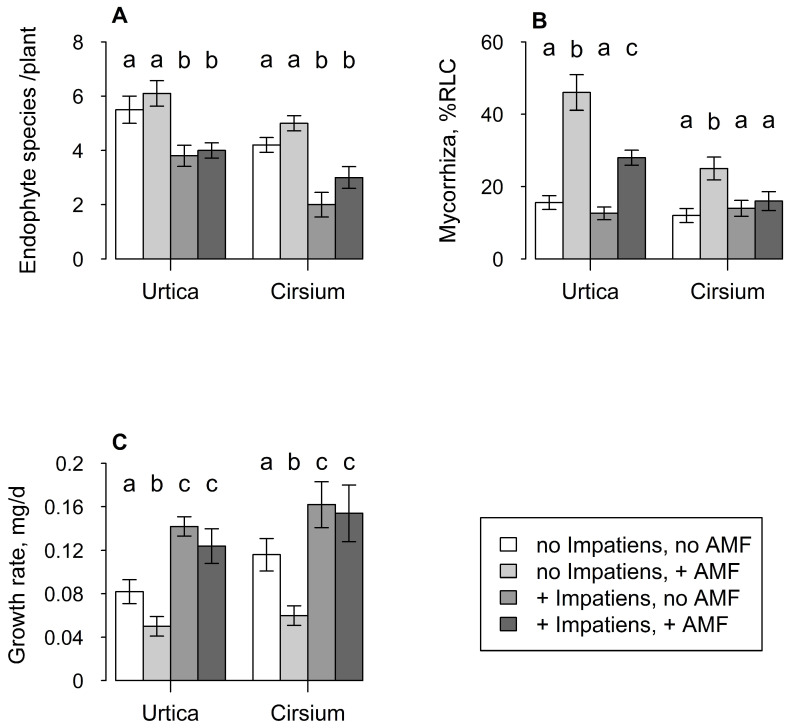
(**A**) Mean number of endophyte fungal species per plant of *Urtica dioica* and *Cirsium arvense* when grown with or without *I. glandulifera* and with or without addition of arbuscular mycorrhizal fungal inoculum. (**B**) Mean percent root length colonised by AM fungi of *U. dioica* and *C. arvense* grown in the same treatments. (**C**) Mean larval growth rate over 28 d of *Mamestra brassicae* larvae reared on leaves of *U. dioica* and *C. arvense* grown in the same treatments. Vertical lines represent ± one standard error. Lowercase letters above bars indicate pairwise differences between means.

The presence of *I. glandulifera* reduced endophyte number per plant in both *U. dioica* (z = 18.3, df = 1, *p* < 0.001) and *C. arvense* (z = 30.7, df = 1, *p* < 0.001) (Figure 5A). Addition of AM fungi had a weak effect of increasing endophyte fungi per plant in *U. dioica* (z = 5.6, df = 1, *p* < 0.05) but no effect was seen in *C. arvense* (Figure 5A).

Addition of AM inoculum increased colonisation levels in *U. dioica*, but of more interest was the fact that the presence of *I. glandulifera* mitigated this response (*F*_1,36_ = 11.3, *p* < 0.01) (Figure 5B), leading to a significant interaction between presence of *I. glandulifera* and addition of AM fungi (*F*_1,36_ = 5.7, *p* < 0.05). While presence of *I. glandulifera* did not affect endophyte fungal numbers in *C. arvense*, there was an interaction between the treatments (*F*_1,36_ = 4.3, *p* < 0.05), because addition of AM inoculum only increased colonisation levels when *I. glandulifera* was absent (Figure 5B).

Growth rate of *Mamestra brassicae* larvae was increased on both *U. dioica* (*F*_1,36_ = 29.6, *p* < 0.001) and *C. arvense* (*F*_1,36_ = 12.1, *p* < 0.01) when their host plants were grown in the presence of *I. glandulifera* (Figure 5C). However, addition of AM inoculum had no overall effect on growth rate in either plant species, and there were no interactions between the treatments.

## 3. Discussion

These experiments have revealed novel mechanisms by which an invasive plant competes with native vegetation and thereby reduces native plant diversity. *I. glandulifera* appears to manipulate the microbial associates of competing native plants, reducing their colonisation levels by AM fungi and the infection of their leaves by foliar endophytic fungi. The reduction in these beneficial fungi levels appears to make some native plants more susceptible to antagonists. Thus, *I. glandulifera* is a successful invader and reducer of native plant diversity, not only through competitive effects, but also through more subtle indirect effects on microbial communities. 

In this study, mycorrhizal fungal inoculation decreased the biomass of balsam when it was grown alone, but tended to increase it when it was grown in both inter- and intraspecific competition. In previous work, the Symbio inoculum has proven to be consistently antagonistic to *I. glandulifera* [18,36]. Furthermore, there appears to be a negative relation between colonisation levels in the field and plant size when the plant is invasive in the UK, but not where it is native, in the Himalayas [17]. The explanation for this antagonism most likely lies in an unbalanced carbon-for-nutrient trade [37]; the remarkable growth rate of this plant means that its root system must be highly efficient at nutrient uptake. This suggests that it derives relatively little nutrient benefit from the symbiosis, while still providing a good supply of carbon to the fungi. The reduction in growth caused by mycorrhizal inoculation in balsam plants without competition is therefore consistent with previous studies. However, plants in the field do not grow in isolation, and thus it is most intriguing that those experiencing competition did not show mycorrhizal-induced reductions in growth. This situation is not unique; the biomass of the invasive annual *Centaurea melitensis* (Maltese star-thistle) was reduced by AM fungi when grown alone, but increased when grown in competition with a perennial grass [38]. This was attributed to the formation of a common mycorrhizal network (CMN) between the plants, which was parasitised by the invader. The formation of a CMN is thought to be an important aspect of plant invasion and the resulting loss of native plant diversity [20]. Nutrient transfer can occur through the network to the invader [39], though the fungi do show preferential effects; for example, nitrogen (N) is most likely to be transferred to hosts that offer the most carbon in return [40,41]. This likely explains why the outcome of an invasive plant entering a CMN can be positive or negative, determined by the diversity of soil resources and the relative nutrient demands and carbon provision of the fungi and the plants involved [42]. It may also explain why *I. glandulifera* is not antagonised by AM fungi in its native range, where it is considerably smaller and the fungi likely to be different species to those in UK soils [17]. 

It was encouraging that the addition of mycorrhizal inoculum increased colonisation of *I. glandulifera* and the native species above that of background levels. However, this did not seem to benefit the native species, and balsam plants were not smallest in the mycorrhizal and interspecific competition treatments, thereby failing to uphold our first hypothesis. Indeed, the addition of AM fungi appeared to reduce the growth of *H. lanatus,* but had no effect on *P. lanceolata* when in competition with *I. glandulifera.* If *I. glandulifera* did connect to a CMN in the competition pots, then any transfer of nutrients to balsam plus increased mycorrhizal status of *H. lanatus* could account for its reduced growth [43]. It Is thought that there is a curvilinear relation between mycorrhizal colonisation levels and the benefit that a plant derives [44,45], and the increased colonisation levels of *H. lanatus* could have tipped the carbon–nutrient balance in favour of the fungi. Meanwhile, *P. lanceolata* is regarded as a mycorrhizal-dependent plant that responds strongly to the fungi [46]. This greater affinity for AM fungi may have benefited *P. lanceolata,* enabling it to compete more effectively with *I. glandulifera.*

In field situations, balsam may reduce the development of the AM fungal network in soil [47], yet it still forms a sparse association with the fungi [2]. Native plants such as *P. lanceolata* grown in soil previously occupied by *I. glandulifera* then show reduced growth [48]. The results presented here suggest that the reduction in AM fungi enables the plant to compete effectively with itself and other species, leading to the development of continuous monocultures. Such an arrested succession caused by soil community manipulation by an invasive species has been reported once before in sand dune systems [49]. *I. glandulifera* is one of the most problematic invasive species across Europe in reducing native plant diversity [50] and it would appear to have a suite of traits that makes it so successful. Its aggressive growth and competitive dominance occur when it competes with native species for light and nutrients [51], while exerting allelopathic effects on competitors [8]. Furthermore, it can cause changes in soil chemical properties, such as ammonium (NH^4+^) and nitrate (NO^3−^), which has detrimental effects on the biomass and performance of neighbouring native species [48,52]. In addition, we have shown here that it can manipulate the microbial associates of neighbouring natives, rendering them less competitive. 

In the first experiment, the addition of AM fungi had no effect on endophyte species richness in *I. glandulifera* or the native plants, contrary to our hypothesis. Furthermore, competition with *I. glandulifera* had no effect on endophytes in the native species. This may be because the experiment was performed in a glasshouse, rather than in the field. The availability of local inoculum sources can be a key factor in determining the endophyte infection levels in plants [29], and here, endophytes were of sporadic and variable occurrence. However, the fact that some endophytes were found only when AM fungi were added while others disappeared with AM inoculation does suggest that there are interactions between the fungal groups within the plant. It has recently been found that the addition of the Symbio inoculum to *I. glandulifera* can increase or decrease the abundance of certain endophytes [36]. Clearly, the fungal identity and experimental location are likely to be two critical factors determining the outcome of future experiments involving these fungi. 

The effect of *I. glandulifera* on indigenous AM fungal communities in the field manifested in a reduction in colonisation of the two native plants, *U. dioica* and *C. arvense* when growing near balsam, upholding our second hypothesis. Similar disruption of native plant mycorrhizal status by an invasive species has been reported before, though the magnitude of the effects depends upon the identity of the fungal and plant species concerned [53]. Perhaps of more interest is the novel discovery that the presence of balsam also reduced foliar endophyte infection levels in these neighbouring plants, further upholding our second hypothesis. To our knowledge, the only previous study of endophytes in an invasive plant and its neighbours quantified the degree of species overlap in the invader (*Ageratina adenophora,* Crofton weed or Mexican devil) and native plants in China [54]. This showed that the invader could harbour latent pathogens of native plants, but that there were distinct differences in their communities. These unspecialised endophytes are ubiquitous in nature, and it was long thought that plants are random interceptors of airborne species, leading to similar communities within different, co-occurring plant species. However, this has been shown not to be the case, with endophyte community composition being determined by many factors, including the identity of the plant, its age, the identity of the fungi, soil nutrients and the presence of other microbes such as AM fungi [29]. 

To this list, we can add the identity of a neighbouring species, though the mechanism by which *I. glandulifera* reduces infection levels of neighbours is unclear. We hypothesised that the competitive effect of balsam might reduce neighbouring plant size, leading to a smaller leaf surface area available for infection. This hypothesis appeared to be upheld, and it may have critical implications for the invasive success of this plant. A previous study found that smaller plants of *Leucanthemum vulgare* harboured fewer endophytes, but such a relationship was not seen in *C. arvense* [30]. Instead, the mycorrhizal status and soil N content have been shown to influence endophyte infection in the latter species [55]. Thus, the effects of *I. glandulifera* may be indirect; alteration of soil nutrient levels or reduction in AM fungal colonisation of *C. arvense* may lead to lower endophyte infection levels, as AM fungal presence has been shown to increase leaf infection levels of some fungal species [18]. In particular, AM fungi can increase infection levels of *C. acutatum* in *I. glandulifera* [36]. It may be no coincidence that this fungus was only detected in *U. dioica* and *C. arvense* when these plants were growing well away from *I. glandulifera.* If a similar effect occurs with other fungi, this could lead to higher endophyte infection in the more mycorrhizal plants growing far from *I. glandulifera.* Two recent studies involving *I. glandulifera* [18,36] appear to be the only current examples of AM fungal effects on unspecialised endophytes in forbs [56], though there are a few examples of AM fungi altering foliar endophyte performance in grasses. Notwithstanding the fact that clavicipitaceous endophytes in grasses are taxonomically and biologically different to the fungi in forbs [29], the presence of AM fungi can enhance or reduce levels of *Epichloe coenophiala,* dependent upon the identity of the mycorrhizal fungal species [57].

The detrimental effect of *I. glandulifera* presence on endophytes in neighbouring plants was confirmed in our manipulative study. This is important, as it shows that differences found in the field are unlikely to be due to chance differences in the spatial abundance of AM fungi [58]. The presence of *I. glandulifera* also determined whether inoculation with AM fungi enhanced colonisation levels or not. This was particularly evident in *C. arvense,* where inoculation only increased AM fungal colonisation when *I. glandulifera* was absent. This provides further evidence for the antagonistic effect of this invasive species on AM fungal abundance. However, of the most interest was the fact that the presence of *I. glandulifera* led to effects on the growth rate of the insect that was feeding on leaves of *U. dioica* and *C. arvense.*

We found that AM fungi reduced herbivore growth, but only when *I. glandulifera* was absent, upholding our third hypothesis. Furthermore, the growth rate was increased when host plants had been grown in the presence of *I. glandulifera.* Currently, we have no clear explanation for this entirely novel discovery, but suggest that the effects may be due to the reduction in endophytes in *U. dioica* and *C. arvense.* Both unspecialised endophytes in the leaves of forbs and AM fungi in the roots can reduce the growth of this generalist chewing insect [59,60], but these results suggest that the endophyte effect on insects is stronger than the mycorrhizal one. Unspecialised endophytes in leaves are thought to act as plant bodyguards, having antagonistic effects on a range of insect species [33]. These effects are likely due to the array of chemicals that these fungi produce or induce within their host plants [61]. Therefore, the presence of *I. glandulifera* reduces the protective effect of these fungi in native plants, rendering them more susceptible to herbivores. This would further enhance the competitive success of *I. glandulifera,* and by weakening native plant growth through manipulation of microbial associates, local plant diversity would be reduced. 

We have previously suggested that adding mycorrhizal inoculum, such as the Symbio product, to field soils might enable native plants to compete more effectively with *I. glandulifera* [18,36]. Although in the present study the inoculum failed to reduce balsam growth in the competition treatments, the results presented here do not necessarily contradict this suggestion. If the appropriate species of AM fungi can be found, their addition might overcome the antagonistic effects of balsam, and increase endophyte diversity (and thus resistance) of native plants. However, before such a suggestion can become reality, a careful examination of the effects that different species of fungi in the inoculum exert on balsam and the effects of inoculation on the indigenous mycorrhizal community must be undertaken. Commercial inocula very often fail to establish in field conditions, often due to competitive effects with local indigenous species [62]. Indeed, commercial inocula have been found to benefit invasive species, while reducing growth of native plants from American grasslands [63]. A detailed analysis of the fate of AM species in the inoculum and the effects on the composition of indigenous communities is clearly needed, yet such analyses are very rarely carried out in mycorrhizal studies [64]. However, the results presented here suggest that the rewards could be high, through direct and indirect methods of weakening balsam growth, while simultaneously enhancing the growth and competitive ability of native plants by increasing their endophyte assemblages.

## 4. Materials and Methods

### 4.1. Experiment One—Effect of Direct Competition with I. glandulifera on Two Native Species 

In September 2017, mature *I. glandulifera* seeds were collected from a wild population at Harmondsworth Moor in Middlesex, UK (51.48° N, −0.48° W) and stored for six months at 4 °C. The seeds were surface-sterilised in 5% sodium hypochlorite, cleaned in sterile distilled water (SDW), and placed on a moist filter paper at 4 °C. The germinating seeds were sown into 20 L pots containing John Innes No. 3 compost (Westland Horticulture, Huntingdon, UK). The application of AM inocula involved the commercial mycorrhizal inoculant from Symbio (Wormley, Surrey, UK) containing *Claroideoglomus etunicatum, Funneliformis mosseae, Gigaspora margarita, Glomus deserticola, Gl. monosporus, Rhizophagus aggregatus, R. clarum*, and *R. irregularis* as used in [18]. The product is named ‘granular mycorrhizae’ and is referred to as Symbio throughout this paper. We applied 2 g of Symbio, which is the recommended rate of inoculation for herbaceous plants, as a layer in a hole prepared for the seedlings. Control plants received 2 g of steam-sterilised inoculum (two cycles at 120 °C for 1 h). Each control also received 15 mL microbial filtrate of live inoculum, in sterile water, passed through a 38 μm membrane, to remove all mycorrhizal propagules, following [18].

The two native plant species in this study frequently co-occur with *I. glandulifera* in the field; these were Ribwort plantain, *Plantago lanceolata*, and Yorkshire fog grass, *Holcus lanatus* [17,48]. The former plant is a perennial forb which can flower in its first year. Mature plants have a strong root association with mycorrhizal fungi and a short, thick rhizome. Meanwhile, the latter species, *H. lanatus*, is a perennial grass that grows in humid climates, most commonly found on fertile soils in meadows, pastures, and rough grassland, and is also mycorrhizal [65,66]. 

A completely randomised design with all the possible pairs of native plant species and *I. glandulifera* were used in the study of interspecific competition: (*I. glandulifera* × *P. lanceolata*) and *(I. glandulifera* × *H. lanatus).* Two *I. glandulifera* plants per pot (equivalent to a common field density of 30 m^−2^ [2]) were grown as an intraspecific competition treatment, as well as balsam grown singly without competition as the control. In the interspecific competition treatment, two *I. glandulifera* and one plant of *P. lanceolata* or *H. lanatus* were grown in a pot, with and without Symbio inoculum, and placed in a glasshouse for nine weeks. All seedlings of *I. glandulifera* and the native species were of identical size when transplanted. There were eight treatments, each with five replicates, for a total of forty plants. The plants received 300 mL of water twice daily. Plant parameters (height and dry shoot biomass) of *I. glandulifera* were recorded prior to flowering to conform to the Animal and Plant Health Agency license, which does not allow flowering or escape of seeds into the wild. Dry shoot biomass of native species was also recorded. As described in Section 4.4 and Section 4.5, root and leaf samples were collected for AM and endophytic fungal analysis when plants were harvested.

### 4.2. Experiment Two—I. glandulifera Effects on Microbial Associates of the Native Species 

The two field sites at either end of the 125 km transect through southern England, described in [31], were used, namely the campus of Royal Holloway University of London, Egham, Surrey, UK (51.43° N, −0.56° W), and River Stour, Zeals, Wiltshire (51.08° N, −2.31° W). At each location, plants of *I. glandulifera*, *Urtica dioica*, and *Cirsium arvense* were excavated for measurement of arbuscular mycorrhizal colonisation (Section 4.4) and foliar endophyte presence (Section 4.5). *U. dioica* and *C. arvense* were selected, as these were the commonest native plants present in both sites. The average stand density of *Impatiens glandulifera* at Royal Holloway was 28 plants m^−2^, while at Zeals it was 34 m^−2^. At each site, 10 asymptomatic plants of *U. dioica* and *C. arvense* were sampled from within dense stands of *I. glandulifera* and also at least 100 m away from the invaded area. Within the stands, each plant of *U. dioica* and *C. arvense* was growing less than 0.5 m from the nearest *I. glandulifera* (which was also sampled), but at least 5 m from its nearest conspecific. Note that the plants of *I. glandulifera* studied here were different individuals to those sampled in [31]. 

### 4.3. Experiment Three–Effect of Competition with I. glandulifera on Plant Microbial Associates 

Rhizomes of *U. dioica* and *C. arvense* were excavated in winter 2016 from natural populations in a meadow at Royal Holloway, where *I. glandulifera* was absent. Previous examination of the roots of these plants in autumn produced mycorrhizal colonisation levels of 18.5 ± 4.8% and 16.8 ± 3.1%, respectively. Pots of volume 20 L were lined with a 38 µ membrane, filled with John Innes compost (as above) and one similar-sized rhizome per pot, planted in January 2017. At planting, 2 g of Symbio was added to the planting hole around each rhizome in half of the pots, and sterilised inoculum + filtrate was added as the control treatment as described in Section 4.1. In April, when shoots from rhizomes were becoming apparent, two seedlings of *I. glandulifera* at the first true leaf stage were planted into half of the pots, to stimulate a density of 30 plants m^−2^. Pots were sunk into field soil in a randomised block design consisting of four treatments for each focal plant species (i.e., *U. dioica* or *C. arvense*): with or without *I. glandulifera* and with or without AM fungal inoculum. Pots were rotated weekly to avoid growth of mycorrhizal hyphae into the pots [67]. Plants were grown for 11 weeks, then were removed from the field before flowering and placed in a glasshouse. Three first instar larvae of the polyphagous moth *Mamestra brassicae* (cabbage moth) were placed on each focal plant and each experimental unit placed inside a fine black muslin bag. Larvae were allowed to develop for four weeks. Average larval weight per plant was determined and growth rate calculated using the formula of van Geffen et al. [68]. Plants were then excavated and the roots of *U. dioica* and *C. arvense* examined for mycorrhizal colonisation (Section 4.4) and foliage sampled for endophyte presence (Section 4.5).

### 4.4. Arbuscular Mycorrhizal Fungi (AMF) Colonisation 

Each plant’s root material was collected for analysis of AM fungal colonisation. The roots were removed from the compost and thoroughly cleaned under running water to remove all soil particles. For root staining, the roots were divided into pieces of about 10 mm length and stored in 70% ethanol. The root staining method followed Vierheilig et al. [69]. Roots were rinsed in tap water to remove the ethanol, and each sample was placed into an individual biopsy processing cassette. At 80 °C for 25 min, the cassettes were submerged in a beaker containing 10% potassium hydroxide (KOH). The cassettes were then removed and rinsed for 10 min under running tap water. The cassettes were placed back into the water bath for 30 min after being submerged in a beaker containing a staining solution (84.4:15:0.6, sterile distilled water (SDW): 1% hydrochloric acid: Quink blue pen ink). The roots were then mounted on a slide in SDW, sealed with nail polish, and examined under a microscope. AM fungal colonisation was measured using the cross-hair eyepiece method of McGonigle et al. [70]. The percentage root length colonisation (RLC) was evaluated by counting 100 intersections with root sections where the presence of intraradical hyphae, vesicles, and arbuscules was recorded, at a magnification of 400×. This process was repeated for all root samples. 

### 4.5. Endophyte Isolation 

Three leaves (bottom, middle, and top) from each plant in each treatment were taken for endophytic fungal assessment. The surface sterilisation method III by Schulz et al. [71] was modified by cutting two round fragments from each leaf using a sterilised hole punch, each approximately 6 mm in diameter. The fragments were submerged in 100% ethanol for 30 s, washed in SDW, immersed in 4.7% sodium hypoclorite (NaOCl) (4.7% *v*/*v*: 4.7 mL NaOCl in 100 mL SDW) for 1 min, submerged in 100% ethanol for another 30 s, and then rinsed 4 times separately with SDW. To prevent bacterial contamination, the fragments were placed abaxial-surface down onto potato dextrose agar (PDA) that was supplemented with 80 mg L^−1^ streptomycin sulphate and 60 mg L^−1^ penicillin G.

The fragments were pressed on several PDA plates prior to plating in order to examine the efficacy of the surface sterilisation process. The 90 mm plates were kept at room temperature in a plastic box and parafilm-sealed to prevent contamination. Single isolations of each endophyte growing from the sterilised leaf were set up on potato carrot agar (PCA) plates. Upon sporulation, fungal material such as conidia, conidiophores, and mycelia were mounted on slides in erythrosin stain, then were identified morphologically by B. C. Sutton. Meanwhile the sterile cultures were sent to the Microbial Identification Service, Centre for Agriculture and Bioscience International (CABI), for molecular identification as described in Currie et al. [31]. For each fungal species, the endophyte isolation frequency (IF) was calculated by dividing the number of isolates of that fungal species per plant by the sum of all isolates of that fungal species in that plant [30]. 

### 4.6. Statistical Analysis 

All of the statistical analyses were conducted using R 4.0.2. Prior to analysis, plant height and dry shoot biomass were tested for normality and residual plots were examined. Percentage data were subjected to the logit transformation prior to analysis [72]. All plant growth data that violated the assumptions were transformed with square root or logarithmic transformations. 

In experiment one, a two-way factorial ANOVA was conducted with mycorrhizal and competitive factors as the main effects to examine the effect and interaction of these factors on Himalayan balsam performance. The height, weight, and RLC percentage of each plant in each pot were used to define *I. glandulifera* performance, and if there were two plants in each pot, the mean of similar parameters was calculated. The performance of native plants (dry shoot biomass and RLC percentage) was analysed using a one-way ANOVA with mycorrhizal presence as the main effect. Similar analytical techniques—two-way ANOVA for *I. glandulifera* and one-way ANOVA for native plants—were used to examine variations in endophyte IF of each fungal species between treatments.

In experiment two, differences in the numbers of endophyte species per plant in *U. dioica* and *C. arvense* when near to and distant from *I. glandulifera* were examined with a Poisson generalised linear model structure, using a log link function, following checking for overdispersion. Differences in mycorrhizal colonisation of the two species near to and distant from *I. glandulifera* were examined using ANOVA following the logit transformation.

In experiment three, differences in the number of endophytes per plant for each species was also examined with a Poisson GLM, employing presence of *I. glandulifera* and AM fungi as main effects. Similarly, differences in mycorrhizal colonisation were examined with two-factor ANOVA following logit transformation. Effects of *I. glandulifera* and mycorrhizal presence on larval growth rate were examined with a general linear model, after checking for normality and examination of residuals.

## 5. Conclusions

*I. glandulifera* forms continuous high-density populations in its invasive range, seriously reducing native plant diversity. Although AM fungi reduce growth of the plant at low densities, the biomass of this plant is greatest when it is in competition with native species. This may be due to its parasitisation of the common mycorrhizal network, reducing the mycorrhizal benefit for native plants. Furthermore, the plant manipulates microbial associates of neighbours, reducing mycorrhizal colonisation of their roots and foliar endophyte infection levels of their shoots. This renders these plants more susceptible to antagonists such as herbivorous insects, further weakening their competitive ability.

## Data Availability

The raw data sets are available from the corresponding author upon reasonable request.

## Supplementary Materials

The following supporting information can be downloaded at: https://www.mdpi.com/article/10.3390/plants12071552/s1, Table S1: Isolation frequency (%) mean of each endophyte species in *Impatiens glandulifera* across treatments. Table S2: Isolation frequency (%) mean of endophyte species in native plants. Table S3: Isolation frequency (%) mean of each endophyte species across all 10 sampled plants in *Impatiens glandulifera, Urtica dioica*, and *Cirsium arvense* at Royal Holloway campus. Table S4: Isolation frequency (%) mean of each endophyte species across all 10 sampled plants in *Impatiens glandulifera, Urtica dioica*, and *Cirsium arvense* at Zeals, Wiltshire, UK.
