# Supplementary material for: The Invasive Plant Impatiens glandulifera Manipulates Microbial Associates of Competing Native Species
Source: Plants (Basel). 2023 Apr 4;12(7):1552. doi: 10.3390/plants12071552 (PMC10096542; doi:10.3390/plants12071552)
Supplement: Supplementary file 1 [file plants-12-01552-s001.zip › plants-2307753-supplementary.pdf]

Table S1 Isolation Frequency (%) mean of each endophyte species in *Impatiens glandulifera* across treatments. +PL referred to competition with *Plantago lanceolata* while +HL competition with *Holcus lanatus*. +/-AM were mycorrhizas present/absent plants.

Endophyte species	Single		Intraspecific		Interspecific +PL		Interspecific +HL	
	+AM	-AM	+AM	-AM	+AM	-AM	+AM	-AM
Acremonium incoloratum	0	0	6.67	0	0	0	0	0
Alternaria alternata	0	0	20	0	0	0	0	0
Chaetomium elatum	0	0	0	20	0	0	0	0
Cladosporium cladosporioides	0	0	0	0	0	0	0	20
Cladosporium oxysporum	20	0	0	20	0	0	0	10
Colletotrichum acutatum	0	0	3.33	0	0	0	0	0
Penicillium spp.	0	0	10	0	0	0	0	10

Table S2 Isolation Frequency (%) mean of endophyte species in native plants. +/-AM were mycorrhizal present/absent plants.

Endophyte species	*P. lanceolata*		*H. lanatus*	
	+AM	-AM	+AM	-AM
Acremonium incoloratum	0	0	5	0
Arthirinium state *Apiospora montagnei*	0	8.33	0	0
Cladosporium oxysporum	8.33	16.67	0	5
Colletotrichum acutatum	0	8.33	0	0
Exophiala sp.	20	0	15	15

Table S3 Isolation Frequency (%) mean of each endophyte species across all 10 sampled plants in *Impatiens glandulifera*, *Urtica dioica* and *Cirsium arvense* at Royal Holloway campus. 'near' indicates plants growing close to *I. glandulifera*,; 'far' indicates plants growing at least 100 m distant from *I. glandulifera*. See section 2.2, main text.

Endophyte species	*I. glandulifera*	*U. dioica*		*C. arvense*	
		near	far	near	Far
Absidia spinosa	0	0	0	3.3	16.7
Acremonium zonatum	0	0	2.9	0	0
Acremonium fusidioides	0	9	0	0	15.5
Alternaria alternata	5	0	7.9	0	22

Alternaria tenuissima	0	0	0	14.5	20
Arthinium arundinis	0	0	0	5	3.33
Chaetomium bostrychodes	2.5	0	14.3	0	0
Chaetosphaeronema hispidulum	0	0	34.1	0	0
Cladosporium cladosporioides	25	0	16.7	10.5	10
Cladosporium oxysporum	33.33	14.3	19.4	8.3	13.1
Colletotrichum acutatum	50	0	25	0	33.3
Colletotrichum dematium	0	0	19.4	0	0
Gliocladium roseum	0	0	0	0	0
Penicillium spp. B	0	11.8	20.3	26.6	0
Periconiella mucunae	0	0	0	0	0
Pseudofusidium sp.	0	19.7	20	0	0
Phoma tropica	0	7.1	10.5	0	10.5
Phoma eupyrena	0	11.7	0	0	0
Phomopsis subordinaria	0	9	29.4	0	12.3
Septofusidium elegantulum	0	11.7	34.5	0	0
Trichoderma koningii	0	0	0	5.3	25
Xylohypha nigrescens	0	0	0	0	0

Table S4 Isolation Frequency (%) mean of each endophyte species across all 10 sampled plants in *Impatiens glandulifera*, *Urtica dioica* and *Cirsium arvense* at Zeals, Wiltshire, UK. 'near' indicates plants growing close to *I. glandulifera*,; 'far' indicates plants growing at least 100 m distant from *I. glandulifera*. See section 2.2, main text.

Endophyte species	*I. glandulifera*	*U. dioica*		*C. arvense*	
		near	far	near	far
Absidia spinosa	0	0	0	0	0
Acremonium zonatum	0	0	10	0	0
Acremonium fusidioides	0	0	0	12.5	22.5
Alternaria alternata	10	5	25	25	33.3
Alternaria tenuissima	0	0	0	0	14.5
Arthinium arundinis	0	0	0	0	0
Chaetomium bostrychodes	0	0	15	10	12.5

Chaetosphaeronema hispidulum	0	0	0	0	22.5
Cladosporium cladosporioides	12.5	5	10	5	12.5
Cladosporium oxysporum	22.5	12	22.4	25	20
Colletotrichum acutatum	15	0	33.3	0	15.5
Colletotrichum dematium	0	0	0	0	10
Gliocladium roseum	0	0	20	5	25
Penicillium spp. B	0	0	14	0	0
Periconiella mucunae	0	0	0	0	10.8
Pseudofusidium sp.	0	3.33	12.5	0	0
Phoma tropica	0	2.5	5.5	2	25
Phoma eupyrena	0	0	0	0	0
Phomopsis subordinaria	0	6	20	0	0
Septofusidium elegantulum	0	5	15	0	0
Trichoderma koningii	0	0	0	10	33.3
Xylohypha nigrescens	0	0	0	0	2.8
